# Deciphering the Hypoxia-immune interface in esophageal squamous carcinoma: a prognostic network model

**DOI:** 10.3389/fonc.2023.1296814

**Published:** 2023-12-12

**Authors:** Jie Hu, Qilong Liu, Bi Feng, Yanling Lu, Kai Chen

**Affiliations:** ^1^ Department of Medical Oncology of The Eastern Hospital, The First Affiliated Hospital, Sun Yat-Sen University, Guangzhou, China; ^2^ Department of Gastroenterology of The Eastern Hospital, The First Affiliated Hospital, Sun Yat-Sen University, Guangzhou, China

**Keywords:** esophageal squamous cell carcinoma (ESCA), hypoxia, weighted gene co-expression network analysis (WGCNA), immune infiltration, prognostic biomarkers

## Abstract

**Introduction:**

The rapid progress and poor prognosis of the exercise of esophageal squamous cell carcinoma (ESCA) bring great challenges to the treatment. Hypoxia in the tumor microenvironment has become a key factor in the pathogenesis of tumors. However, due to the lack of clear therapeutic targets, hypoxia targeted therapy of ESCA is still in the exploratory stage.

**Methods:**

To bridge this critical gap, we mined a large number of gene expression profiles and clinical data on ESCA from public databases. First, weighted gene co-expression network analysis (WGCNA) and functional enrichment analysis were performed. We next delved into the relationship between hypoxia and apoptotic cell interactions. Meanwhile, using LASAS-Cox regression, we designed a robust prognostic risk score, which was subsequently validated in the GSE53625 cohort. In addition, we performed a comprehensive analysis of immune cell infiltration and tumor microenvironment using cutting-edge computational tools.

**Results:**

Hypoxia-related genes were identified and classified by WGCNA. Functional enrichment analysis further elucidated the mechanism by which hypoxia affected the ESCA landscape. The results of the interaction analysis of hypoxia and apoptotic cells revealed their important roles in driving tumor progression. The validation results of the prognostic risk score model in the GSE53625 cohort obtained a good area under the receiver operating characteristic (ROC) curve, and the risk score was independently verified as a significant predictor of ESCA outcome. The results of immune cell infiltration and tumor microenvironment analysis reveal the profound impact of immune cell dynamics on tumor evolution.

**Conclusion:**

Overall, our study presents a pioneering hypoxiacentered gene signature for prognostication in ESCA, providing valuable prognostic insights that could potentially revolutionize patient stratification and therapeutic management in clinical practice.

## 1 Introduction

Esophageal squamous cell carcinoma (ESCA), an aggressive malignancy characterized by its late diagnosis and poor prognosis, has become a major health concern globally. The incidence and mortality rates of ESCA are a reflection of an evolving public health landscape, shaped by socio-economic and lifestyle changes that have altered dietary patterns and risk factor exposures (1). ESCA stands as the eighth most commonly diagnosed cancer and the sixth leading cause of cancer-related mortality worldwide, making it a disease of significant epidemiological weight (2). The latest global cancer statistics reveal a sobering picture: in 2018, ESCA accounted for over half a million new cases and an almost equal number of deaths, representing 3.2% of all cancer cases and 5.3% of cancer deaths respectively (2). Notably, China bears a disproportionate burden of ESCA, with nearly 70% of the world’s cases reported there, bringing into focus the regional disparities in cancer epidemiology (3, 4).

The high fatality rates associated with ESCA can largely be attributed to its typically asymptomatic nature in early stages, leading to delayed diagnoses when surgical interventions are less viable due to advanced metastasis. The five-year survival rate languishes below 20%, a statistic that has seen little improvement over the years, highlighting the critical need for innovative treatment strategies and more effective management approaches (5, 6).

The tumor microenvironment of ESCA, particularly under hypoxic conditions, is a critical determinant of tumor progression and treatment resistance. Within this niche, apoptotic cells—those undergoing programmed cell death—can significantly alter tumor behavior and response to treatment, presenting a potential target for therapeutic intervention. Despite the nascent stage of immunotherapy in the ESCA treatment arsenal, there are promising signs that immunomodulatory approaches could enhance outcomes, especially for patients exhibiting certain biomarkers such as PD-L1 (7, 8). This optimism is bolstered by clinical trials showing favorable responses to immunotherapy in subsets of ESCA patients, suggesting a new frontier in personalized cancer care (9).

The role of the tumor microenvironment extends beyond the confines of tumor cells, encompassing a complex network of cellular and extracellular components that support and enable tumor survival, growth, and metastasis (10, 11). Hypoxia is a hallmark of the tumor microenvironment that significantly contributes to this network. It arises when rapidly proliferating tumor cells outstrip their blood supply, resulting in a low-oxygen environment that activates a cascade of molecular events through hypoxia-inducible factors (HIFs). These factors are pivotal in orchestrating a range of cellular adaptations that not only facilitate tumor survival and progression but also contribute to genetic instability and resistance to conventional therapies (12, 13). The adverse impact of hypoxia on treatment outcomes has been well-documented, solidifying its status as a key prognostic factor in ESCA and other solid tumors (14).

In parallel, the immune landscape within the tumor microenvironment has emerged as a critical influencer of tumor evolution, with hypoxia exerting a profound effect on both innate and adaptive immune responses. These interactions offer new insights into potential immunotherapeutic targets and necessitate a deeper understanding of the hypoxia-immune interface in ESCA (15, 16). This study aims to bridge this knowledge gap by leveraging bioinformatics tools to construct a robust hypoxia-related gene model that can predict prognosis and guide therapeutic interventions for ESCA. Utilizing comprehensive datasets from TCGA and hypoxia gene sets from MSigDB, we applied WGCNA to identify key hypoxia-associated genes and performed functional enrichment analysis to decipher the underlying biological mechanisms. The prognostic significance of these genes was evaluated using LASSO-Cox regression to create a risk scoring system, which was externally validated in the GSE53625 cohort. Our exploration also extended to the analysis of immune cell infiltration and the immune microenvironment using advanced algorithms such as ESTIMATE, xCell, CIBERSORT, and TIMER, providing a panoramic view of the immune dynamics in ESCA and their implications for patient survival and therapy optimization.

## 2 Materials and methods

### 2.1 Data collection

Data was collected from the TCGA repository (https://portal.gdc.cancer.gov/), This dataset consisted of RNA sequencing data from 162 ESCA samples and 11 normal paracancerous tissues, accompanied by clinical information pertaining to ESCA patients (17). Hypoxia-related genes were retrieved from the msigdb.org website. Through our search, we identified gene sets M5891, M10508, and M641, comprising 311 hypoxia genes in total (18). Additionally, we established a validation cohort (GSE53625), which included the RNA sequencing of 358 patients and corresponding survival data from the GEO repository (https://www.ncbi.nlm.nih.gov/geo/) (19).

### 2.2 Co-expression network construction

The WGCNA method was used to investigate gene set expression. The WGCNA R package was utilized to develop and modularize various gene networks. This was done through several main phases: identifying significant outliers through sample clustering, establishing co-expression networks using automated networks, and detecting modules through hierarchical clustering and dynamic tree cutting functions. Module Membership (MM) and Gene Significance (GS) were calculated to establish associations between modules and clinical features. Hub modules were identified based on the highest Pearson MM correlation, with an absolute P-value of 0.05. An MM value greater than 0.8 and a GS value exceeding 0.2 were considered indicators of strong module connectivity and clinical relevance, respectively. The gene data of the relevant module underwent further scrutiny.

### 2.3 Consensus clustering

We implemented consensus clustering to discern unique pyroptosis-related trends associated with hypoxia-related gene expression utilizing the k-means approach. Through the consensus clustering algorithm and “ConsensusClusterPlus” package, the cluster count and corresponding robustness were determined. To ensure the classification’s solidity, we executed 1,000 iterations.

### 2.4 GO and KEGG pathway enrichment analysis

To delineate the functional significance of differentially expressed genes in esophageal squamous cell carcinoma (DEOSG), we employed Gene Ontology (GO) and Kyoto Encyclopedia of Genes and Genomes (KEGG) pathway analyses utilizing the “clusterProfiler” (20), “ggplot2” (21), and “enrichplot” (22) R packages. This approach illuminated the biological processes, molecular functions, and pathways the DEOSGs are implicated in. Visual representations were crafted for clarity using “ggplot2” and “enrichplot”, with a statistical significance threshold set at a P-value of less than 0.05.

### 2.5 Identification of hypoxia-related gene prognostic markers

Univariate Cox regression analysis was used to identify prognosis-associated genes. Furthermore, we used LASSO Cox regression to select robust independent prognostic indicators for ESCA, considering a p-value < 0.05 to be significant. We calculated risk scores using the following equation: Risk scores are calculated by summing the product of coefficients [Coef(i)] and mRNA expression levels [X(i)] for each gene in the module. Where ‘n’ is the number of genes in the module. If the coefficient [Coef(i)] is negative, it indicates a protective effect of the gene on patient survival. Conversely, if the coefficient [Coef(i)] is positive, it indicates an adverse survival pattern associated with the gene. The TCGA-ESCA tumor samples were divided into two categories based on their risk level: high-risk and low-risk. This classification was determined using the median cut-off value. The Kaplan-Meier (K-M) method was used to analyze and evaluate the prognostic significance of these two groups. In addition, the receiver operating characteristic (ROC) curve was used to evaluate the accuracy and precision of the classification, specifically measuring sensitivity and specificity.

### 2.6 Evaluation of immune cell infiltration and generation of tumor microenvironment scores

We used the established method of immune cell estimation analysis, CIBERSORT, to determine the ratios of 22 types of immune cell subtypes based on the TCGA cohort. We used TIMER to assess the abundance of six immune cell types. To understand the level of immune cell infiltration in different risk categories, we used xCell and ESTIMATE algorithms to generate estimation scores, immune scores, and stromal scores to examine additional tumor microenvironment. Results with a P value < 0.05 were advanced for further study. We used Spearman correlation to verify the correlation of risk scores with tumor microenvironment scores. A 2-way ANOVA analysis was performed to check the association of risk scores with immune infiltration subtypes.

### 2.7 Cell culture

The EC109 ESCA cell line and the HEEC normal esophageal cell line obtained from the American Type Culture Collection (Manassas, USA) were cultured in RPMI-1640 medium supplemented with 10% fetal bovine serum. Additionally, a mixture of penicillin-streptomycin was added to confer dual resistance. All cell cultivation was performed under moist conditions at a temperature of 37°C and 5% CO2.

### 2.8 Western blot

The total protein treated with RIPA buffer was quantified using BCA (Thermo, PA, USA). After this step, proteins were separated using a 12% SDS-PAGE technique, subsequently transferred onto a PVDF membrane. Then, the membrane was blocked using milk powder and left overnight to be immunostained with primary antibodies against CD59 (ab133707, 1:1000), IGFBP2 (ab188200, 1:1000), KRT15 (ab262484, 1:10000), BIK (ab52182, 1:500), SDC4 (ab74139, 1:500), ARPC4 (ab217065, 1:2000), TPD52 (ab181260, 1:1000), GAS2L1 (ab246924, 1:1000), RANGAP1 (ab92360, 1:5000) and GAPDH (ab8245, 1:1000) at 4°C. Following incubation with the secondary antibody, we captured fluorescent signals using a chemiluminescence system (Pierce, Thermo, PA, USA).

### 2.9 RNA purification and qRT-PCR

We used TRIzol reagent to extract total RNA from the collected samples (cells). The concentration, purity, and integrity of the collected total RNA were evaluated by a UV spectrophotometer and agarose gel electrophoresis. Subsequently, reverse transcription of cDNA was performed using a high capacity cDNA kit (Applied Biosystems, USA). The RNA sample of 1 µg was used together with random primers during the synthesis process. The amplification process was performed using a one-step SYBR PrimeScript RT-PCR kit and an ABI 7500 PCR system. The amplification protocol and conditions were followed according to the instructions provided with the kit. PCR amplification was performed for 40 cycles, with each sample repeated in three wells, and the entire experiment was replicated three times. In this study, GAPDH was used as the internal mRNA reference. We used the 2-△△ct method for data analysis. The primer sequences are shown in **Table 1**.

**Table 1:** Primer sequences.

ID	Upstream primer (5’ -3’)	Downstream primer set (5’ -3’)
CD59	CGTCAGGTGTGTATTGGGCT	GGGCACACAGTAGGTTCTCC
IGFBP2	TGCAGACAATGGCGATGACC	GGTGCTGCTCAGTGACCTTC
KRT15	AGAAATCTGAATTCCTATTGCAGGAGA	CCCTGAAAGCTTAGACCGAGGGACCCT
BIK	GACCATGGAGGTTCTTGGCA	AGGCTCACGTCCATCTCGTC
SDC4	CAAGGTGTCAATGTCCAGCA	AGAGGATGCCCACGATGC
ARPC4	GAAAGGGGTCCAAGCAGTGT	TGGTGGTGCAATACACGGAA
TPD52	GGAAGAGGAGCAGGAAGAGC	GATGACTGAGCCAACAGAG
GAS2L1	CATCTGGTGGGAAAGGGGTC	GGAGGACTTACGCCATGCAA
RANGAP1	CAGGCTTTCGCTGTCAACC	GCAGCATCCCTCTTGATTTCAC
GAPDH	GCTGGCGCTGAGTATGGAGT	CACAGTCTCTTGGTGATGG

### 2.10 Statistical analysis

All data processing and analysis was performed in R Studio (version 4.1.1). We used independent samples t-tests and Mann-Whitney U-tests to compare the two groups of continuous variables, chi-square or Fisher exact tests to analyze the statistical significance between the two groups of categorical variables, and Pearson correlation analyses to estimate correlation coefficients between different genes. All statistical p-values were two-sided and we considered statistically significant when p < 0.05.

## 3 Results

### 3.1 A weighted co-expression network was created, and through an appropriate method, key modules were identified

**Figure 1:**
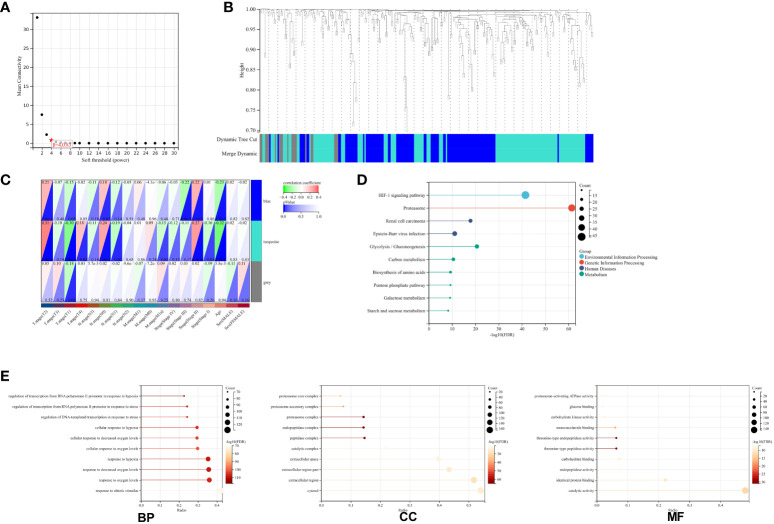
Network Construction and Gene Module Associations. **(A)** Soft-threshold power analysis illustrating the influence on network connectivity. **(B)** Dendrogram of all genes clustered based on a dissimilarity measure (1-TOM) with assigned module colors. **(C)** Heatmap depicting the correlation between module eigengenes and clinical traits of ESCA. **(D)** KEGG pathway enrichment for key genes in the blue and turquoise modules highlighting involved biological pathways. **(E)** GO term enrichment for blue and turquoise module hub genes, categorized into biological processes (BP), cellular components (CC), and molecular functions (MF).

Upon exploring the MSigDB database for hypoxia gene sets, we identified three sets: M5891, M10508, and M641. Integration of these sets resulted in a total of 311 hypoxia-related genes (**Supplementary Table 1**). These genes were then integrated with 161 TCGA-ESCA samples, creating a matrix for WGCNA analysis. The analysis yielded three modules (**Figures 1A, B**). To examine the correlation between these modules and clinical parameters, we conducted Pearson’s test, which revealed significant correlations between the blue and turquoise modules and T stage, N stage, as well as the clinical stage of ESCA patients (**Figure 1C**). In order to gain a better understanding of the functions of genes within these two modules, we performed GO and KEGG analyses. The KEGG enrichment analysis showed significant associations between these genes and key biological pathways, including proteasomes, the HIF-1 signaling pathway, and the glycolysis/gluconeogenesis pathway (**Figure 1D**). Additionally, the GO enrichment analysis indicated that genes within both modules are involved in important biological processes such as RNA polymerase II promoter transcriptional regulation in response to hypoxia and response to reduced oxygen levels (**Figure 1E**).

### 3.2 Consensus clustering identifies two ESCA clusters with different hypoxic status

**Figure 2:**
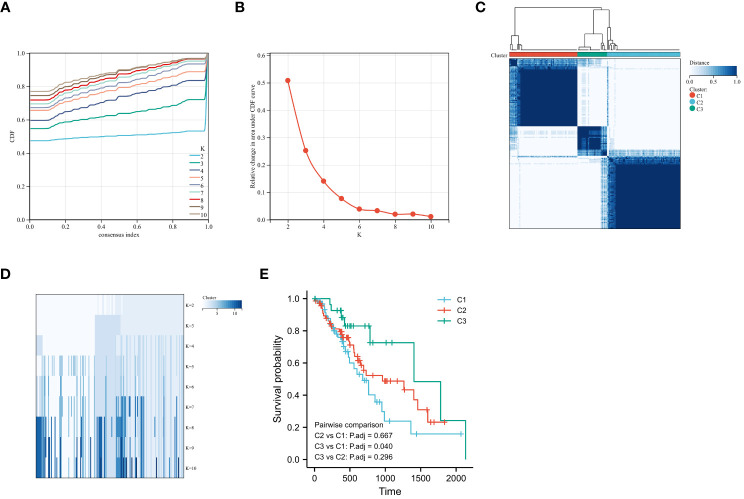
Consensus Clustering and Survival Analysis for ESCA. **(A)** Cumulative distribution function (CDF) for consensus clustering. **(B)** Delta area plot determining the optimal number of clusters. **(C)** Consensus matrix heatmap for k=2 clusters. **(D)** Item-consensus tracking plot for varying cluster numbers (k=2 to k=10). **(E)** Kaplan-Meier curves comparing overall survival rates among identified ESCA subgroups.

To determine the hypoxic status of 161 TCGA-ESCA tumor samples, consensus clustering was performed according to the expression similarity of 205 hypoxia-associated gene features. With K=3, the CDF curve had a flatter mid-section (**Figures 2A-D**). Consequently, two subgroups, named cluster1 (n=64), cluster2 (n=69), and cluster3 (n=28), were determined. The OS rate was notably higher in the C3 group in contrast to the C1 group (**Figure 2E**).

### 3.3 Impact of hypoxic states on immune cell infiltration in ESCA

**Figure 3:**
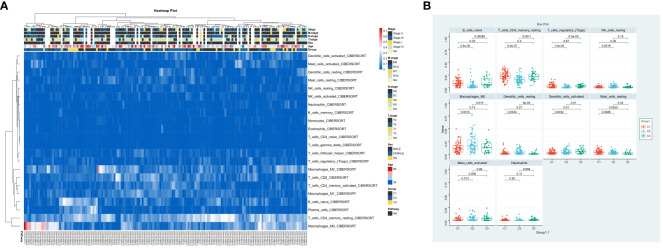
Immune Landscape Profiling in ESCA Subgroups. **(A)** Bar chart comparing the relative levels of immune cell infiltration across three ESCA patient clusters. **(B)** Profile of immune cell infiltration delineated into 10 immune cell types.

**Figure 4:**
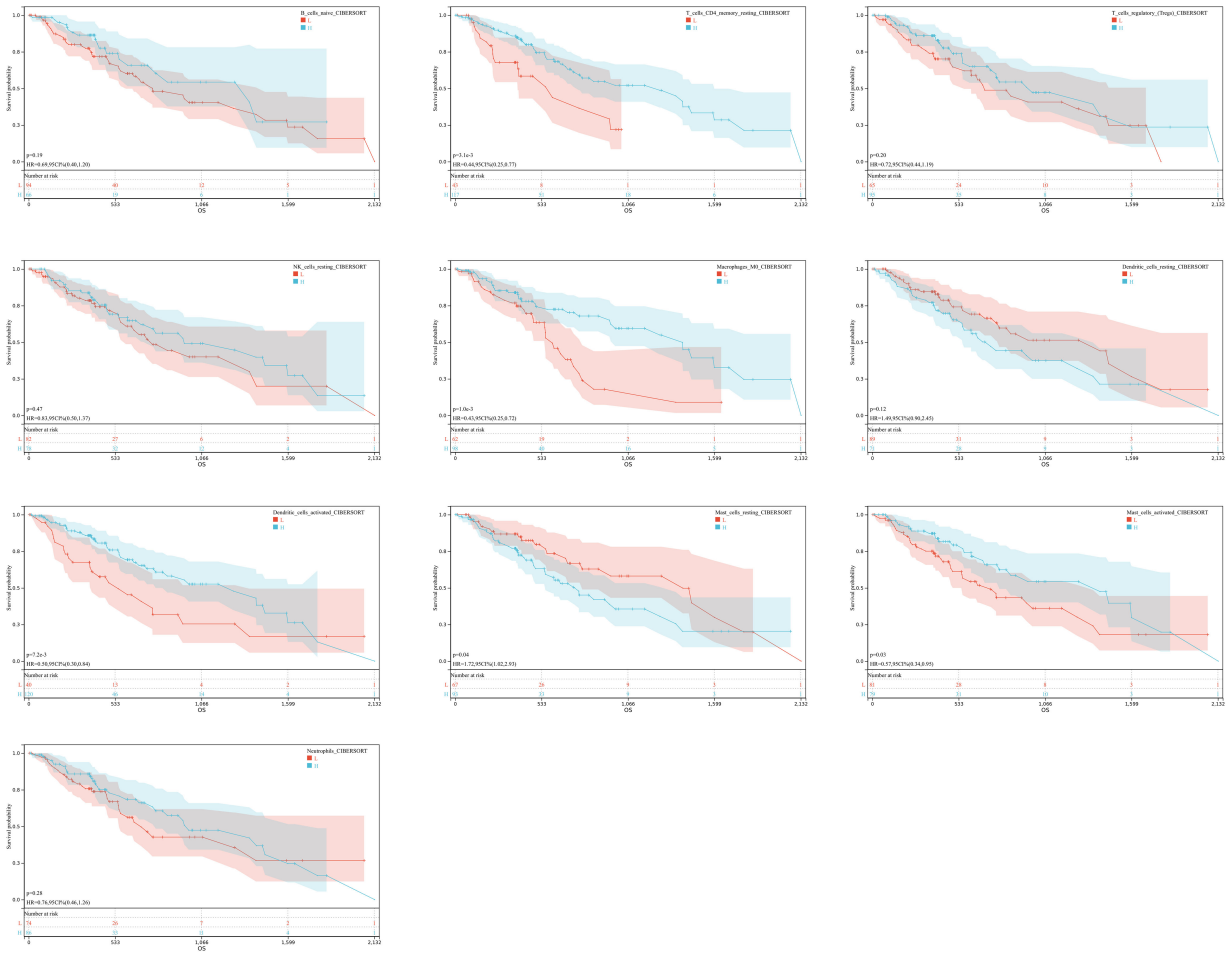
Immune Expression-Survival Correlation. A graphical representation correlating immune cell expression with patient survival rates in ESCA.

In order to understand the role of hypoxia in tumor progression and its impact on the immune response, we utilized the xCell algorithm in the R platform to analyze immune cell subtypes. We observed variations in the abundance of several cell types, including B cells naive, resting memory CD4 T cells, Tregs, resting NK cells, M0 Macrophages, resting dendritic cells, activated dendritic cells, resting mast cells, activated mast cells, and neutrophils among the three groups (**Figures 3A, B**). Out of the 22 immune cell types evaluated, these specific cell types showed differential abundance. Additionally, our K-M analysis revealed that high expression of T cells CD4 memory resting, Macrophages M0, activated dendritic cells, and activated mast cells, as well as low expression of resting mast cells, were associated with an unfavorable prognosis (**Figure 4**).

### 3.4 Developing and validating prognostic features of hypoxia-associated genes

**Figure 5:**
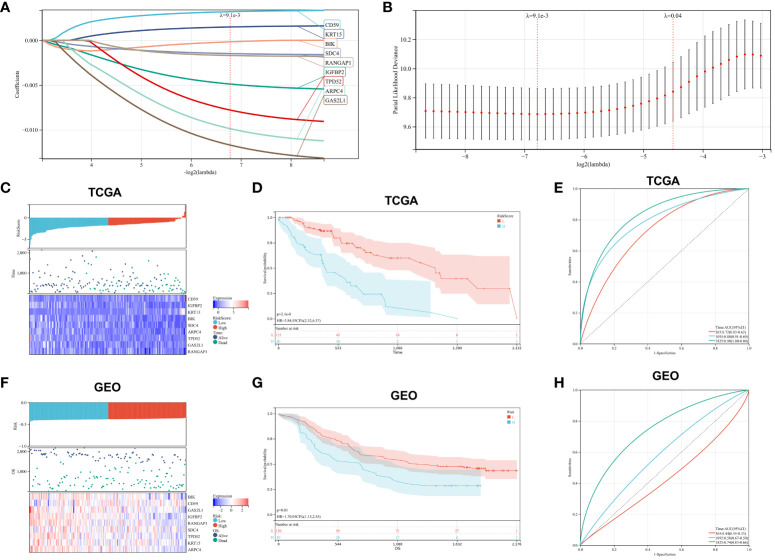
Prognostic Marker Construction and Validation. **(A, B)**. LASSO Cox regression for identification of optimal prognostic gene markers. **(C)**. Risk score distribution, patient survival status, and expression heatmaps for key hypoxia-associated genes from TCGA. **(D)**. Kaplan-Meier survival analysis correlated with risk scores from TCGA data. **(E)**. ROC curves validating the prognostic accuracy for 1-, 3-, and 5-year outcomes in TCGA. **(F–H)**. Corresponding analysis for GEO dataset, mirroring the structure and content of panels **(C–E)**.

In our retrospective study, we delved into the prognostic significance of hypoxia-related genes in ESCA by conducting univariate Cox regression and LASSO regression analyses on a refined cohort of 205 pivotal genes identified within the blue and turquoise modules. The meticulous analytical process culminated in the recognition of nine hypoxia-associated genes significantly correlated with patient outcomes, namely CD59, IGFBP2, KRT15, BIK, SDC4, ARPC4, TPD52, GAS2L1, and RANGAP1. Leveraging the coefficients derived from LASSO regression, we established a risk score formula and subsequently stratified patients into high-risk or low-risk categories, with the median risk score serving as the demarcation (**Figures 5A–C**). The Kaplan-Meier survival analysis underscored a markedly improved overall survival (OS) in the low-risk group (P<0.001) (**Figure 5D**). Additionally, the efficacy of our risk model in prognosticating OS was substantiated through ROC curve analysis, yielding robust AUC metrics at 1-year (0.72), 3-year (0.80), and 5-year (0.90) intervals, thus highlighting the model’s discernment capabilities (**Figure 5E**). To extend the model’s validity, an external dataset (GSE53625) was employed. Concordance with TCGA-ESCA data was affirmed via Kaplan-Meier analysis, where the survival disparity predicated on the risk score reaffirmed the model’s consistency and predictive prowess (**Figure 5F**). In the external cohort, the low-risk group notably outpaced the high-risk group in survival outcomes (P<0.05), and the model’s predictive accuracy was echoed with AUCs of 0.73, 0.77, and 0.83 for 1-year, 3-year, and 5-year survival rates, respectively (**Figures 5G, H**). These findings assert the model’s applicability and reliability across diverse datasets, thus reinforcing its potential utility in clinical prognostication for ESCA.

### 3.5 Correlation of risk score, clinical features, and immune score in ESCA patients

**Figure 6:**
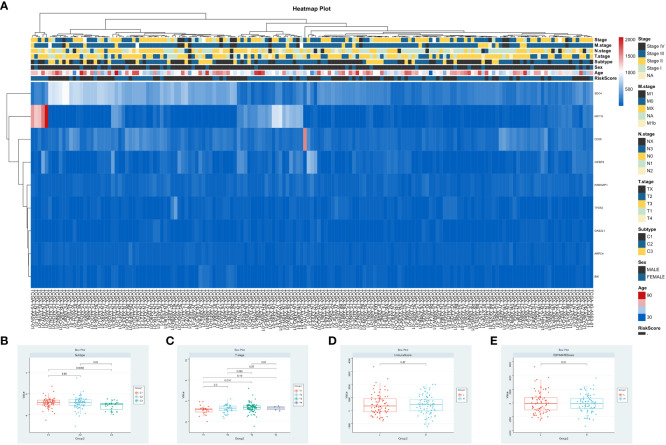
Integrative Analysis of Risk Scores and Clinical Immune Scores. **(A)** Correlation analysis of risk scores, patient clinical attributes, and expression patterns of significant genes. **(B–E)**. Visualization of risk scores in relation to patient clusters, tumor stages (T), Immune Scores, and ESTIMATE Scores.

To investigate the relationship between risk score, clinical characteristics, and cluster subgroups, researchers conducted a heatmap analysis. The analysis revealed that ARPC4, BIK, IGFBP2, SDC4, and TPD52 were present at lower levels in the high-risk group, suggesting their protective role for ESCA patients. On the other hand, KRT15 showed higher expression in the low-risk cohort, indicating its association with increased risk for ESCA patients. Notable differences were observed between the high-risk and low-risk groups in terms of ESCC cluster subtypes (P < 0.001) and T staging (P < 0.05) (**Figure 6A**). Risk scores varied significantly among different groups, with groups C1 and C2 having notably higher risk scores compared to group C3 (P < 0.001, **Figure 6B**), indicating distinct risk profiles within ESCC subtypes. Furthermore, the risk score for T1 stage patients was lower compared to T3 stage patients (P < 0.01, **Figure 6C**). Interestingly, no significant differences were found between the Immune Score, ESTIMATE Score, and risk score (**Figures 6D, E**). These findings emphasize the significant impact of risk scores on the clinical outcomes of ESCC patients.

### 3.6 Correlation between risk score and immune cell infiltration and apoptosis

**Figure 7:**
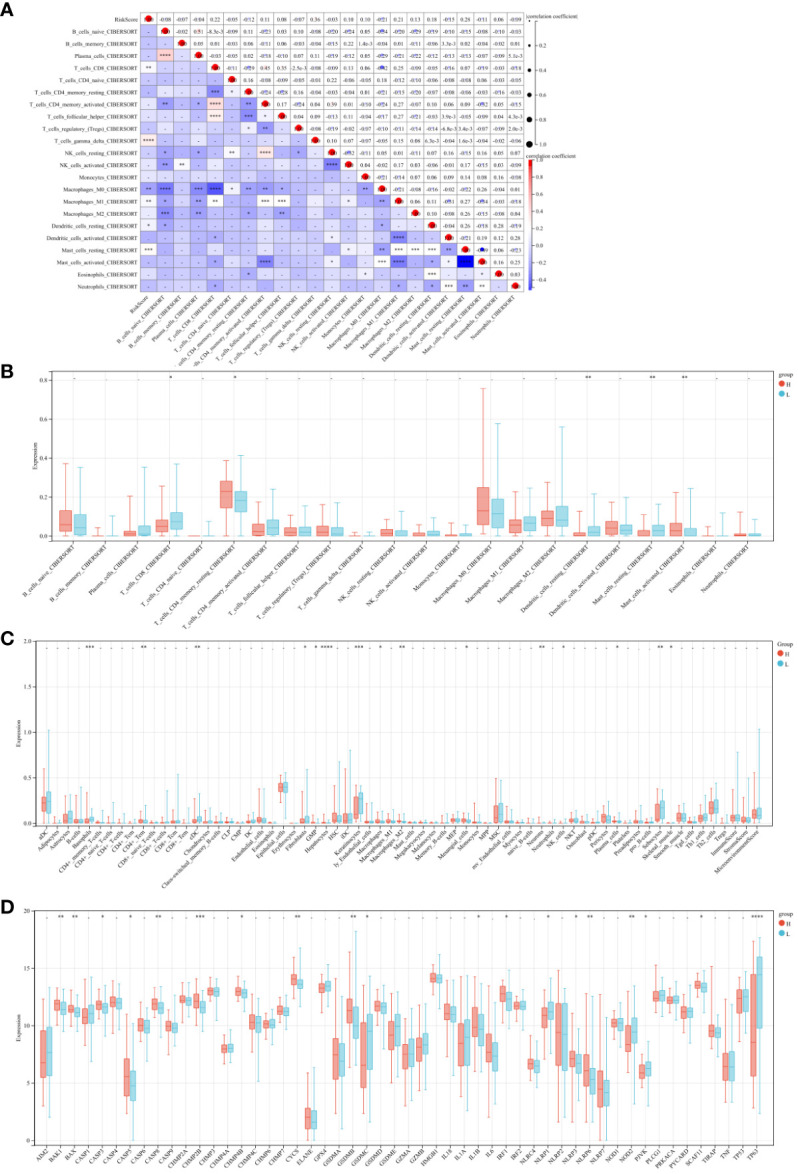
Risk Score and Immune Cell Infiltration Correlation Study. **(A)** Scatter plot examining the relationship between risk scores and 22 types of immune cell infiltrations. **(B)** Comparative analysis of the immune cell infiltration levels between high and low-risk groups. **(C)** Heatmaps depicting the infiltration of 64 immune cell types across risk groups. **(D)** Comparative gene expression analysis of apoptosis-related genes in high versus low-risk groups. * P<0.05, ** P<0.01, *** P<0.001 and **** P<0.0001.

To assess the impact of the 9 central genes on the immune microenvironment of ESCA, we examined the correlation between the risk score and the infiltration levels of 22 types of immune cells (**Figure 7A**). The results indicate a positive association between the risk score and the infiltration levels of T cells CD8, T cells gamma delta, Macrophages M1, Dendritic cells resting, and Mast cells resting (**Figures S1A, B**, **S1D, E**). Conversely, there is a negative correlation between the risk score and the infiltration levels of Macrophages M0 (**Figure S1C**). Furthermore, the levels of CD8 and mast cells were significantly lower in the high-risk score patients compared to the low-risk group (**Figure 7B**). Additionally, we observed that among the 64 immune cells calculated by xcell, the hepatocytes level was the most significant in the high-risk group compared to the low-risk group (**Figure 7C**). Finally, we also compared the expression of apoptotic genes between the high-risk and low-risk groups, which showed a significant increase in the expression of proapoptotic genes and a decrease in the expression of apoptotic genes in the high-risk group (**Figure 7D**). These findings confirm the association between the risk features of the 9 central genes and the immune microenvironment of ESCA.

### 3.7 *In vitro* validation of hub genes

**Figure 8:**
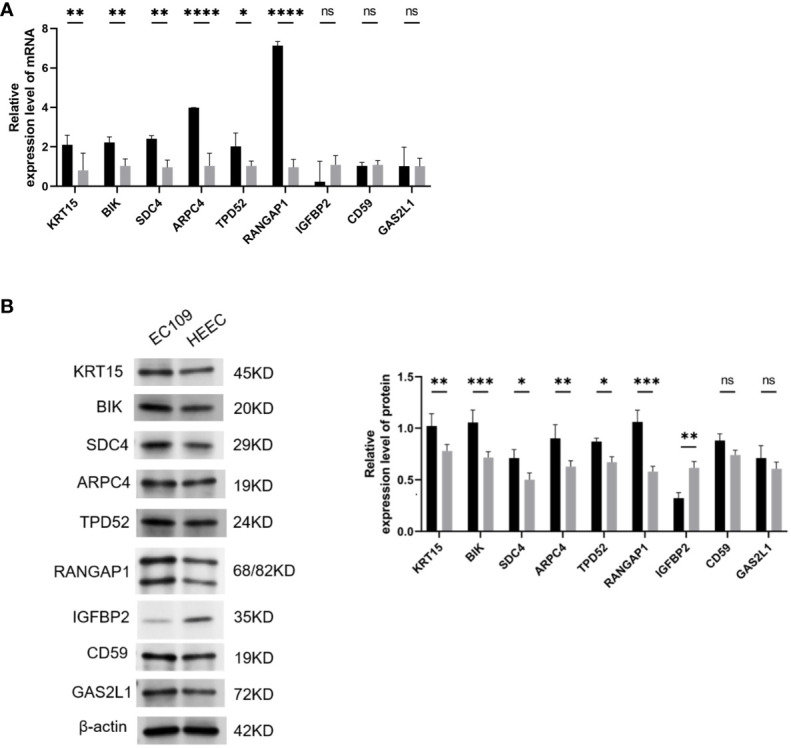
Validation of Hub Gene Expression via qRT-PCR and Western Blot. **(A)** qRT-PCR results showing the mRNA levels of hub genes in ESCA cell lines. **(B)** B. Western blot analysis visualizing the protein expression levels of hub genes in ESCA cell lines. Statistical significance denoted as ns (P > 0.05), *(P < 0.05), **(P < 0.01), ***(P < 0.001), and ****(P < 0.0001). ESCA: Esophageal Squamous Cell Carcinoma. This optimized version focuses on clarity and consistency, ensuring each figure is described succinctly while maintaining all necessary scientific detail and referencing the impact on ESCA research.

In the end of our study, we used qRT-PCR and WB to detect the expression of KRT15, BIK, SDC4, ARPC4, TPD52, RANGAP1, IGFBP2, CD59, and GAS2L1 in ESCA cells. The qRT-PCR and WB results showed that KRT15, BIK, SDC4, ARPC4, TPD52, and RANGAP1 are highly expressed in ESCA cells (P <0.05), while IGFBP2 is expressed at a lower level (P <0.01). No difference was found in the expression of CD59 and GAS2L1 between ESCA and normal cells (P>0.05, **Figure 8**).

## 4 Discussion

ESCC, a common malignancy of the digestive tract, is characterized by an unfavorable prognosis (23). It is a common subtype in our country with poor treatment outcomes and a low survival rate (24). Despite the widespread use of the TNM staging system as a risk stratification tool for ESCC patients (25), it does not always accurately predict patient outcomes. As molecular biology has advanced and our understanding of the immune microenvironment has grown, certain genes have been identified as prognostic markers for ESCC patients (26). In addition, the role of apoptotic cells in the tumor microenvironment has gained attention. Apoptotic cells can influence the tumor microenvironment, especially in the context of hypoxia, by releasing various factors that can modulate immune responses, promote tumor growth, and influence treatment outcomes. However, the immune microenvironment is incredibly complex and varies significantly between tumor types (20). Therefore, we’ve chosen tumor purity as a criterion for evaluating the immune microenvironment. Given the variability in tumor purity and hypoxic conditions between different tumor types, the question remains as to how best to use gene expression profiles to more effectively predict ESCC prognosis.

In recent decades, there have been tremendous advances in molecular research and bioinformatics technology. Molecular biology allows a detailed examination of how variations and co-expression in genes can affect protein functionality and disease progression. This is achieved through enrichment analysis, a process in which genes are classified into groups based on their molecular functions, the biological processes in which they are involved, and the cellular components with which they are associated (21). This analysis helps to understand the roles these genes play in different biological contexts, providing a clearer picture of their significance in the disease process. At the same time, WGCNA has proven to be a valuable tool for exploring relationships between diseases and related phenotypes and highly correlated gene clusters (modules) (22). Numerous studies have used WGCNA to uncover the functions and potential molecular mechanisms of hub genes (27, 28). In our research, we were able to identify 205 hypoxia-related genes using WGCNA. These genes were functionally associated with hypoxia response in GO and KEGG enrichment analyses, including transcriptional regulation of RNA polymerase II promoters in response to hypoxia, response to reduced oxygen levels, and critical biological pathways, including the HIF-1 signaling pathway and the glycolysis/gluconeogenesis pathway. In addition, we performed an assessment of immune cell infiltration, which revealed the significant influence of hypoxia on the immune microenvironment within tumors. Furthermore, the interplay between hypoxia and apoptosis was evident, suggesting that the balance between cell survival and programmed cell death under low oxygen conditions may significantly impact tumor progression and treatment outcomes. Our analysis revealed that there were 10 distinct immune cell subtypes that showed differential expression. Significantly, some of these Subtypes such as memory resting CD4 T cells, activated M0 macrophages, activated dendritic cells and activated mast cells showed high expression levels. In contrast, resting mast cells showed low expression levels. These findings underscore the importance of immune infiltration in the development of ESCC and suggest that hypoxia may influence patient prognosis by affecting immune infiltration.

To validate our hypothesis, we performed Lasso regression and identified nine hypoxia-related genes associated with ESCC prognosis. The results showed that CD59, IGFBP2, KRT15, BIK, SDC4, ARPC4, TPD52, GAS2L1, and RANGAP1 were correlated with ESCC prognosis. CD59 is a cell surface expressed protein that helps protect cells from complement-mediated lysis. Wang et al. (29) found that CD59 expression on red and white blood cells increased significantly after a combination of hypoxia preconditioning and high-altitude training. This suggests a role for CD59 in the regulation of hypoxic conditions. IGFBP2 is one of the major regulators of insulin-like growth factors (IGFs) and can modulate the biological activity of IGFs (30). Studies have shown that IGFBP2 can ameliorate tumor hypoxic conditions by promoting vascular neovascularization in the tumor microenvironment (31). BIK, a member of the BCL-2 protein family, is a pro-apoptotic protein responsible for regulating cell apoptosis. It plays a critical role in many physiological processes and diseases such as cancer and neurodegenerative disorders (32). Research has shown that tumor cells can adapt to hypoxic conditions and resist hypoxia-induced cell death by altering their metabolism and physiological characteristics through the regulation of BIK expression, highlighting the intricate relationship between hypoxia and apoptosis (33). SDC4 belongs to the syndecan protein family and is a cell surface-anchored transmembrane protein. It can interact with many bioactive molecules such as growth factors, chemokines, and cell adhesion molecules, thereby regulating various cellular behaviors and physiological processes (34). Due to its role in cell adhesion and migration, SDC4 can undergo changes under hypoxic conditions. It can upregulate its expression to enhance migration ability and escape the hypoxic environment (35). However, there is a lack of research on the association between KRT15, ARPC4, TPD52, GAS2L1, RANGAP1 and hypoxic conditions. We hope to conduct experimental studies in future research to validate the changes in these genes under hypoxic conditions.

Researchers have developed a hypoxia analysis model to better understand the relationship between hypoxia-associated gene expression and patient prognosis. This model successfully stratified the population into two risk categories, allowing for a more comprehensive assessment of patient outcomes. The study revealed a striking contrast in prognosis between the high-risk and low-risk cohorts. Specifically, individuals in the low-risk cohort had significantly higher survival rates for both ESCC and EAC compared to their counterparts in the high-risk cohort. In addition, the validity of this model was confirmed using external data, further supporting its predictive ability in estimating 3-year and 5-year patient survival. The involvement of hypoxia in the progression of ESCC has been highlighted, as it promotes tumor glycolysis, angiogenesis, cell proliferation, metastasis, and confers resistance to radiation and chemotherapy (36). Hypoxia or hypoxic stress has emerged as a marker of the immune microenvironment. Rapidly proliferating tumors require a substantial supply of nutrients and oxygen, leading to tumor angiogenesis (37). However, this process is typically abnormal and inefficient. Oxygenation of tumor regions close to blood vessels relies on the diffusion gradient relative to intravascular oxygen tension, resulting in hypoxia in more distant regions (38). The presence of tumor hypoxia in ESCC has been validated by studies, and it’s considered a significant risk factor for adverse prognosis in ESCC patients (39). In addition, hypoxia can promote and maintain an immunosuppressive microenvironment (37). It primarily mediates immune suppression by inhibiting T-cell migration into tumor tissue or accelerating T-cell apoptosis (40). In addition, hypoxia can also induce apoptosis in tumor cells, leading to the release of various factors that can modulate immune responses. The balance between hypoxia-induced apoptosis and immune responses can significantly affect tumor progression and treatment outcome. In-depth analysis revealed a correlation between risk scores, clinical characteristics and immune scores, highlighting the impact of the immune microenvironment on ESCA progression. This underscores the potential of the risk score model to assess both patient immune status and prognosis, further highlighting the importance of immune factors in ESCA development and treatment outcomes. The final part of the study involved basic experimental analysis using qRT-PCR and WB detection. It revealed high expression levels of KRT15, BIK, SDC4, ARPC4, TPD52 and RANGAP1 in ESCA cells, with IGFBP2 expressed at low levels. ESCA and normal cells did not differ in the expression of CD59 and GAS2L1. These findings suggest that high expression of KRT15, BIK, SDC4, ARPC4, TPD52, and RANGAP1 and low expression of IGFBP2 may be associated with the initiation and progression of ESCA. The expression patterns of these genes may be useful in diagnosing, predicting, and monitoring treatment response in ESCA and may provide potential therapeutic targets for ESCC.

Despite the significance of these findings, our study has several limitations. First, our prognostic model needs further validation in more cohorts to demonstrate its applicability in different populations. Second, although our study proposes the hypothesis that hypoxia may influence immune infiltration and elucidates its potential mechanism, further experiments are needed to investigate the exact effects and mechanisms. In addition, the role of apoptosis in the context of hypoxia and its impact on the immune microenvironment needs to be further investigated. Finally, our study is based on the analysis of gene expression levels, and further investigation is required to determine the specific functions of these genes and their roles in ESCA.

In conclusion, our study provides a novel perspective and tool for the therapy and prognostic assessment of ESCA, and it has important implications for understanding the pathological mechanisms of ESCA, including the interplay between hypoxia, apoptosis, and the immune microenvironment.

## Data availability statement

The datasets presented in this study can be found in online repositories. The names of the repository/repositories and accession number(s) can be found below: https://www.ncbi.nlm.nih.gov/, GSE53625.

## Ethics statement

Ethical approval was not required for the studies on humans in accordance with the local legislation and institutional requirements because only commercially available established cell lines were used. Ethical approval was not required for the studies on animals in accordance with the local legislation and institutional requirements because only commercially available established cell lines were used.

## Author contributions

JH: Conceptualization, Formal analysis, Writing – original draft. QL: Methodology, Writing – original draft. BF: Software, Validation, Writing – original draft. YL: Visualization, Writing – original draft. KC: Supervision, Writing – review & editing.

## Supplementary material

Supplementary Figure 1 Evaluating the correlation between risk score and the presence of 22 immune infiltrating cells **(A–F)**. Analyzing the relationship between risk score and the infiltration of distinct immune cell categories, encompassing CD8 T cells, gamma delta T cells, M0 Macrophages, M1 Macrophages, resting Dendritic cells, and also resting Mast cells.
