# Supplementary figures and images for: Deciphering the Hypoxia-immune interface in esophageal squamous carcinoma: a prognostic network model
Source: Front Oncol. 2023 Dec 12;13:1296814. doi: 10.3389/fonc.2023.1296814 (PMC10751000; doi:10.3389/fonc.2023.1296814)

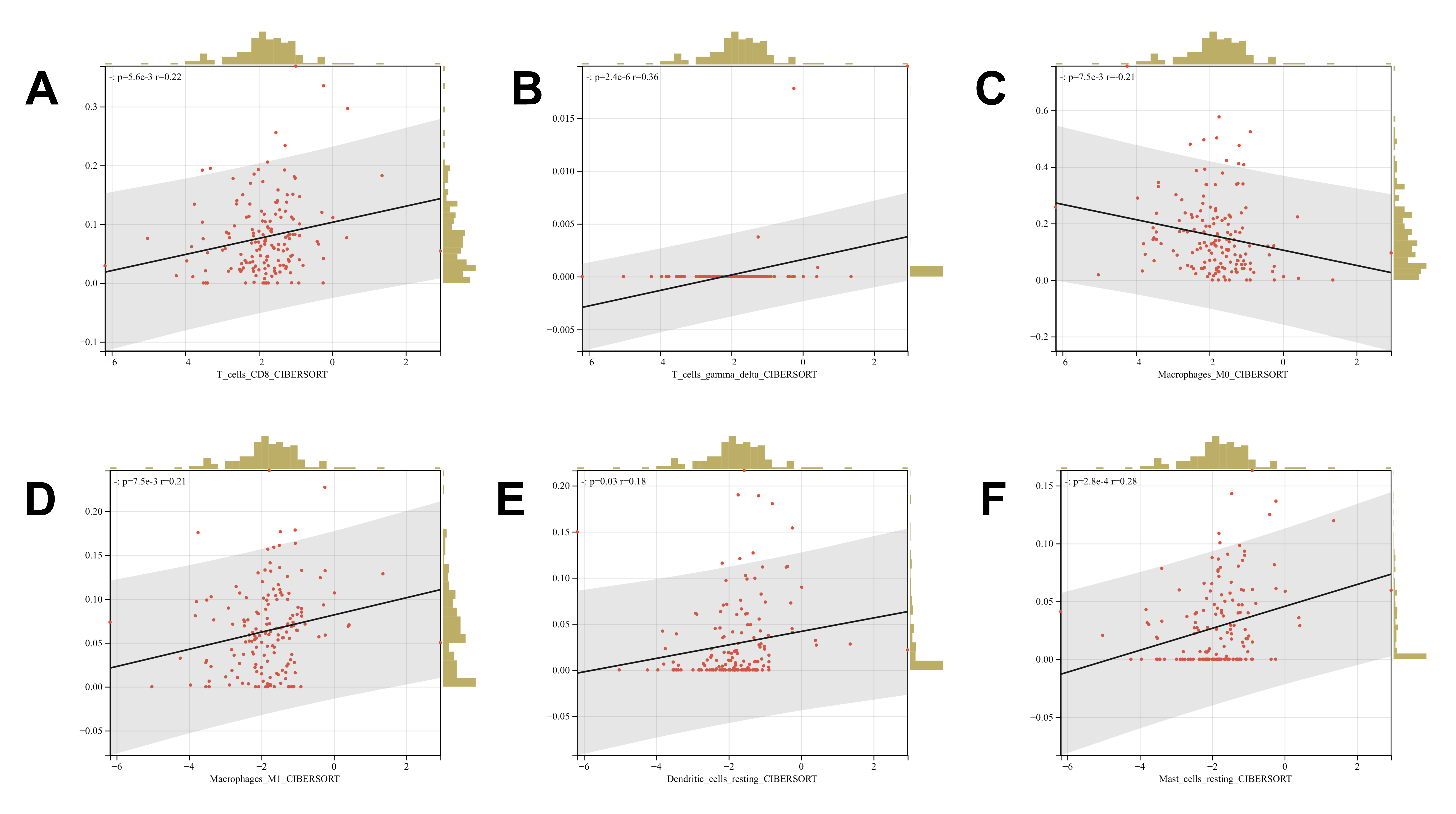

Supplement: Supplementary Figure 1 — Evaluating the correlation between risk score and the presence of 22 immune infiltrating cells (A–F). Analyzing the relationship between risk score and the infiltration of distinct immune cell categories, encompassing CD8 T cells, gamma delta T cells, M0 Macrophages, M1 Macrophages, resting Dendritic cells, and also resting Mast cells. [file Image_1.tif]
