# Supplementary material for: Deciphering the Hypoxia-immune interface in esophageal squamous carcinoma: a prognostic network model
Source: Front Oncol. 2023 Dec 12;13:1296814. doi: 10.3389/fonc.2023.1296814 (PMC10751000; doi:10.3389/fonc.2023.1296814)
Supplement: Supplementary file 2 [file Table_1.docx]

Table S1 hypoxia-related genes ID

| Gene name | | | | | | | | |
| --- | --- | --- | --- | --- | --- | --- | --- | --- |
| ABCF2 | CHST3 | GPI | MT2A | SIAH2 | BNIP3 | EPAS1 | KDELR3 | PKP1 |
| ACKR3 | COL5A1 | GPR87 | MYH9 | SLC25A1 | BNIP3L | ERO1A | KIAA0930 | PLAC8 |
| ACOT7 | COPA | GPRC5A | NAP1L1 | SLC29A1 | BOP1 | ETF1 | KLF6 | PLAUR |
| ADGRG1 | CORO1B | GYG1 | NCLN | SLC2A1 | BRMS1 | ETS1 | KLHL24 | PLIN2 |
| ADM | CXCR4 | GYS1 | NDRG1 | SLC35C1 | BTG1 | EXOSC4 | KPNA4 | PNRC1 |
| AHNAK2 | CYB561 | HCFC1R1 | NDST1 | SLC52A2 | CA12 | EXT1 | KRT15 | PPIF |
| AK3 | DCN | HDLBP | NDUFS6 | SLCO4A1 | CA2 | FAM162A | KRT7 | PRDX5 |
| AK4 | DDIT3 | HIF1A | NFIL3 | SORT1 | CALM1 | FBP1 | LDHA | PRPF4B |
| ALDOA | DDIT4 | HK1 | NR2F6 | SOX9 | CAV1 | FKBP4 | LOX | PXDN |
| ALDOC | DDR1 | HK2 | NUP98 | SPP1 | CAVIN1 | FLNB | LOXL1 | QSOX1 |
| AMD1 | DDX3X | HLA-DRB1 | ODC1 | SPRY1 | CAVIN3 | FOXO3 | LOXL2 | RANGAP1 |
| ANXA2 | DPYSL2 | HNRNPH1 | OGDH | SRM | CCDC86 | FUS | LRRC59 | RBCK1 |
| APEX1 | DSC2 | HSF1 | OLFML2A | STC1 | CCN1 | GAA | LXN | RPP25 |
| ARHGDIA | DST | HSPA4 | P4HA1 | STIP1 | CD59 | GADD45B | MAPK1 | S100A2 |
| ARPC4 | DUSP1 | IDS | PAM | STX3 | CD99 | GAPDH | MAPKAPK2 | S100A4 |
| ATP2A3 | EFNA1 | IGFBP2 | PFKP | SULT2B1 | CDC20 | GAS2L1 | MAZ | S100A6 |
| BAK1 | EFNA3 | IGFBP3 | PGK1 | SUMO3 | CDKN1A | GDF15 | MGAT5 | SDC3 |
| BGN | EIF5A | IGFBP5 | PGM2 | TAGLN | CDKN1B | GJA1 | MIF | SDC4 |
| BHLHE40 | ELF3 | IRS2 | PIM1 | TFAP2C | CDV3 | GLUD1 | MRPS12 | SDF2L1 |
| BIK | ENO1 | JUN | PKM | TFRC | CEMIP | GNA13 | MT-ND5 | SELENBP1 |
| CHKA | GPC1 | MT1E | SERPINE1 | ZMYND8 | XRCC6 | ZFP36 | XRCC5 | WSB1 |
| TGFA | TGFB1 | TGFBI | TGM2 | TIMM23 | TIPARP | TMEM259 | TOMM22 | TPBG |
| TPD52 | TPI1 | TXN | TXNIP | VEGFA | VHL | VIM |  |  |
